# Hyaluronic Acid: A New Approach for the Treatment of Gingival Recession—A Systematic Review

**DOI:** 10.3390/ijerph192114330

**Published:** 2022-11-02

**Authors:** Vini Mehta, Gerta Kaçani, Mohammed M. Al Moaleem, Anwar Abdulkarim Almohammadi, Malak Mohammed Alwafi, Abduljabbar Khalil Mulla, Shahad Owaidh Alharbi, Abdullah Waleed Aljayyar, Etleva Qeli, Çeljana Toti, Agron Meto, Luca Fiorillo

**Affiliations:** 1 Department of Public Health Dentistry, Dr. D.Y. Patil Dental College and Hospital, Dr. D.Y. Patil Vidyapeeth, Pune 411018, India; 2 Department of Prosthetic, Faculty of Dental Medicine, University of Medicine, 1005 Tirana, Albania; 3 Department of Prosthetic Dental Science, College of Dentistry, Jazan University, Jazan 45142, Saudi Arabia; 4 Faculty of Dentistry, University of Ibn Al-Nafis for Medical Sciences, Sana’a 4337, Yemen; 5 College of Dentistry, Taibah University, Al Madinah Al Munawwarah, Medina 41311, Saudi Arabia; 6 Department of Conservative, Faculty of Dental Medicine, University of Medicine, 1005 Tirana, Albania; 7 Department of Dentistry, University of Aldent, 1007 Tirana, Albania; 8 Department of Biomedical and Dental Sciences, Morphological and Functional Images, University of Messina, 98100 Messina, Italy; 9 Multidisciplinary Department of Medical-Surgical and Odontostomatological Specialties, University of Campania “Luigi Vanvitelli”, 80121 Naples, Italy

**Keywords:** gingival recession, Miller class I and II, hyaluronic acid, root coverage

## Abstract

This systematic review aimed to investigate the effectiveness of hyaluronic acid (HA) on the clinical treatment outcomes of patients with gingival recession. A systematic search was performed in PubMed, Cochrane Central Register of Controlled Trials, Embase, Scopus, and Google Scholar for studies up to 15 August 2022. Two reviewers separately selected the papers for eligibility after conducting a thorough search. The study includes randomized controlled clinical trials in which participants were given HA in addition to periodontal treatment surgical procedures. The changes following the treatment protocol were evaluated for complete and mean root coverage as a primary outcome and gingival recession gain as the secondary outcome. Three articles met the eligibility criteria out of 557 titles. In periodontal surgery, HA exhibited better results in complete root coverage and mean root coverage when compared to the control group. Gingival recession reduction, clinical attachment level, and keratinized tissue gain were significantly increased compared to the control groups. However, the comparison presented in the following study might show heterogeneity among the studies and risk of bias in general. Given the scope of this analysis, results suggest that adjunctive treatment with HA gel for root coverage could be clinically beneficial.

## 1. Introduction

Tooth root exposure due to the apical movement of the gingival margin, compared to the cement-enamel junction, is termed a gingival recession [1,2]. Persistent trauma or periodontal disease, anatomic factors of soft tissue (e.g., narrow band of keratinized mucosa), or areas of biofilm development (e.g., inadequately suited dental restorations/crowding) are causes to one or more teeth can be affected by the gingival recession, thus, resulting in tooth sensitivity, hygiene issues, root cavities, and other issues [2,3,4]. As a result, for patients with good dental hygiene, surgical treatment of gingival recession was necessary to reduce these problems. Sub-epithelial connective tissue grafts (SCTG), coronally advanced flaps (CAF), semilunar coronally advanced flaps, laterally positioned flaps, and free gingival grafts have all been used to address gingival recessions [5,6,7,8,9]. The primary downside of the SCTG procedure is the need for donor tissue. In cases with multiple recessions, it necessitates a substantial tissue volume, which causes post-operative pain [10]. Therefore, as a result, biomaterials and alternative grafts have been proposed, including acellular dermal matrix (ADM), enamel matrix derivatives (EMDs), and autologous plasma [11,12,13]. Recently, the role of hyaluronic acid (HA) as a chemotherapeutic agent has been a boon to the advancement in dentistry. HA is a major natural carbohydrate component of the extracellular matrix in many tissues, including the periodontium. The biological effects of HA depend heavily on molecular weight. Hyaluronic acid with molecular weights from 0.4 to 4.0 kDa acts as an inducer of inflammation and has a non-apoptotic property. HA, with a molecular weight of 20–200 kDa, takes part in biological processes such as embryonic development and wound healing. By contrast, high molecular weight hyaluronic acid (>500 kDa) has anti-angiogenic activity and can function as a space filler and a natural immunologic depressant [14]. It has unique physiochemical and biological properties such as viscoelastic, anti-inflammatory, hygroscopic, bacteriostatic, osteoinductive, and anti-edematous properties [15,16,17]. Moreover, numerous trials proved that HA enhances clot formation, induces angiogenesis, promotes osteogenesis, and plays important roles in cell differentiation, adhesion, and migration. HA-binding proteins mediate these to cell surface receptors. Thus, it has been proposed that HA might be a suitable material for both periodontal wound healing and regeneration in periodontal defects. With its application in intra-bony defects, HA has shown promising effects like clinical attachment level (CAL) gain, probing depth reduction, and complete root coverage (CRC) [15,16,18,19,20,21,22]. However, there is no qualitative or quantitative analysis of its efficacy with respect to gingival recession. Thus, this review aimed to systematically investigate the efficacy of HA outcomes on the clinical treatment of patients with labial gingival recession.

## 2. Materials and Methods

The review was conducted according to the Preferred Reporting Items for Systematic Reviews and Meta-Analyses (PRISMA) statement guidelines [23].

### 2.1. Study Registration

The review was registered in the PROSPERO database (the International Prospective Register of Systematic Reviews hosted by the National Institute of Health Research, University of York, Centre for Reviews and Dissemination) on 25 November 2021, according to the guidelines with the identification number CRD42021287145.

### 2.2. Focused Question

Is HA in randomized controlled clinical trials (RCTs) effective in the treatment of gingival recession compared to the control group? Current systematic review objectives are outlined in Table 1.

### 2.3. Search Strategy

Science search engines such as PubMed-MEDLINE, Cochrane Central Register of Controlled Trials, Embase, and Scopus were employed to find articles that could fulfill the study’s objectives. Additional sources, such as IndMed, Google Scholar, and major publications, were combed through, with no language restrictions, from the earliest date until 15 August 2022. The Saudi Digital Library and other ongoing trial registries were searched. For any unpublished studies, contact with the authors was made. Table 2 shows a complete PubMed search strategy that can be modified for additional databases.

#### 2.3.1. Eligibility Criteria

1. RCTs.
2. Studies carried out with no restrictions on age and sex, having gingival recession ≥2 mm.
3. Studies including patients with/without radiographic evidence of bone loss.
4. Intervention/test: Treatment of gingival recession with HA along with coronally positioned flap.
5. Control/Comparison: Treatment of gingival recession with coronally positioned flap alone.
6. Patients who received antibiotic/antifungal therapy, such as metronidazole or anti-hypersensitivity therapy, had any serious illness or malignancies and were receiving treatment for the same were excluded. This review did not include prospective and retrospective observational studies, case reports, letters to editors, reviews, and case series.

#### 2.3.2. Outcome Parameters

The primary outcomes of this study were CRC and mean root coverage (MRC) of the recession. Secondary outcomes measured were gingival recession reduction (GRR), keratinized tissue gain (KT), clinical attachment level (CAL), and follow-up time. Adverse effects such as post-operative complications, discomfort while healing, and root sensitivity were also measured as secondary outcomes.

### 2.4. Screening and Selection

The paper was independently explored by two reviewers, first by the title and abstract. Case reports, letters, and narrative/historical reviews were not included in the search. The papers were selected for full-text reading if keywords were present in the title and the abstract. Papers without abstracts but with titles that were related to this review’s objectives were also selected to screen the full-text eligibility. After selection, full-text papers were read in detail by two reviewers. Those papers that fulfilled all selection criteria were processed for data extraction. Two reviewers manually reviewed all selected article reference lists for additional relevant papers. Disagreements between the two reviewers were resolved by discussion. If a disagreement persisted, the judgment of a third reviewer was considered decisive.

### 2.5. Quality Assessment

Two reviewers independently assessed the risk of bias in each included trial, using the Cochrane Collaboration’s bias assessment technique, with any disagreements addressed by consensus [24]. Risk of bias was performed using software (Review Manager, version 4.2 for Windows, The Nordic Cochrane Centre, The Cochrane Collaboration, Copenhagen, Denmark). The following criteria were marked to access bias in studies “low”, “high”, and “unclear” risks: (a) Random sequence generation, (b) allocation concealment, (c) blinding of participants and personnel, (d) blinding of outcome assessment, (e) incomplete data outcome, (f) selective reporting, and (g) other bias. Low risk was declared if all criteria were declared “low risk”, “high” if at least one criterion was deemed “high risk,” and “unclear” if at least one criterion was deemed “unclear risk” but no other criteria was deemed “high risk.”

### 2.6. Assessment of Heterogeneity

The following were the factors that influenced the heterogeneity of various study outcomes:

1. Concentration of HA.
2. Application of HA.
3. Duration of studies.

## 3. Results

### 3.1. Search and Selection Results

**Figure 1:**
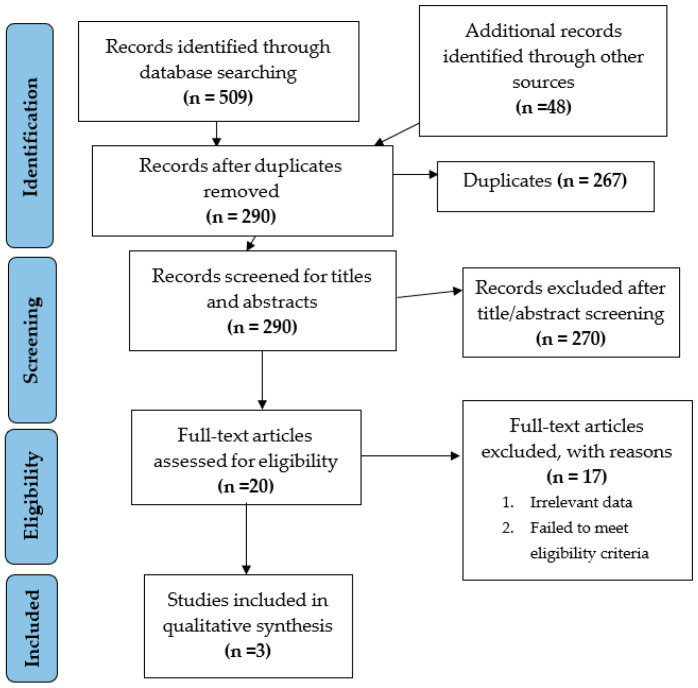
Flowchart summarizing the article selection process (*n*—number of studies).

The electronic search identified 557 unique records using PubMed-MEDLINE, Cochrane CENTRAL, Scopus, Embase, and additional sources (Figure 1). After duplicate removal, 290 records were screened for title/abstract reading and 20 studies were selected for full-text screening. Seventeen studies were excluded after full-text screening. Thus, a total of three studies [17,21,22] that met our inclusion criteria were processed for data extraction. Table 3 gives an overview of the selected studies [17,21,22] and their features.

### 3.2. Characteristics of Included Studies

Table 3 summarizes three trials aimed at the effectiveness of HA in periodontal surgery on patients with Miller class I and II gingival recessions [17,21,22]. A parallel research design was used in one trial, while a split-mouth design and randomized controlled trial were used in the other [17,21,22].

The studies were conducted in 67 patients, and all of them completed the trial. Gracey curettes were used for subgingival debridement in all three investigations. Patients in all these studies were instructed to rinse with 0.12% Chlorhexidine after surgery. Adverse effects like post-operative pain, swelling, complications, and allergies were noted in reviewed studies.

### 3.3. Concentration of HA Application

The method of applying HA and the time interval differed among the trials, but in all three, HA was at least applied once during the therapy procedure. Kumar et al. used Hyaluron gel (0.2% HA, Gengigel, Ricerfarma Pharmacheuticals, Milan, Italy). Nandanwar et al. chose Hyaloss matrix gel (HA, Hyaloss matrix, Meta, Italy), while a cross-linked high-concentrated HA gel (2 mg/mL HA, hyaDENT BG, Bioscience, Germany) was used by Pilloni et al. [17,21,22]. The follow-up period in two studies was 6 months, while the other presented 18 months [17,21,22]. Detailed characteristics and concentration of HA are summarized in Table 4.

### 3.4. Complete and Mean Root Coverage

CRC and mean root coverage (MRC) have shown a significant difference in the included studies [17,22]. According to Kumar et al., when analyzed by Student’s *t*-test after six months of surgery, MRC was presented 68.3% in the study group and 61.7% in thecontrol group [21]. However, complete root coverage was not measured in this aforementioned study. By using Student’s unpaired *t*-test, Nandanwar et al. found a significant increase in CRC (77.7%) for the study group, six months post-operatively, compared to (65.25%) in the control group [22]. Mean root coverage in Nandanwar et al. study was measured 92.8% in the test group and 84% in the control group. Pilloni et al. also concluded that there is a significant increase in complete root coverage, with 80% in the test group and 33.3% in the control group 18 months postoperatively [17].

### 3.5. Gingival Recession Reduction

Gingival recession reduction was found statistically significant in the included studies [17,22]. After six months, increased mean reduction was seen in the gingival recession for the test group (1.0 and 2.55 mm) than in the control group (1.1 and 2.11 mm) [21,22]. Similarly, Pilloni et al. discovered a significant difference between the groups, with the test group experiencing a 2.7 mm reduction in recession compared to 1.9 mm in the control group [17].

### 3.6. Evaluation of CAL

CAL has shown significant improvement for all the trials included in this study. Pilloni et al. reported a statistically significant difference in CAL gain in the test group (*p* = 0.023) and (*p* = 0.011) for the control group [17]. By analyzing through Student’s unpaired *t*-test, Nandanwar et al. concluded that there is a significantly higher CAL gain in the test group (3.03 mm) compared to the control group (2.34 mm) [22]. Kumar et al. [21] observed significant gain in the CAL after 24 weeks post-operatively.

### 3.7. Keratinized Tissue Gain

Heterogeneity was noted among the studies when comparing keratinized tissue gain. Pilloni et al. found no difference in keratinized tissue gain between the two groups (*p* = 0.116) [17]. Using Student’s unpaired *t*-test, Nandanwar et al. noted an increase in keratinized tissue gain of 2.53 mm in the test group compared to 1.96 mm for the control group [22].

### 3.8. Synthesis of Result

Despite the fact that data were extracted in a systematic manner for this review, a meta-analysis was not possible owing to study heterogeneity and inconsistent data.

### 3.9. Quality Assessment

**Figure 2:**
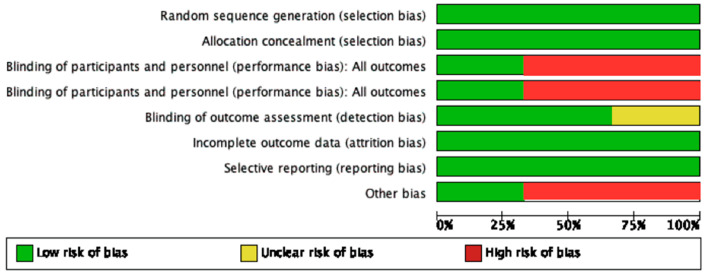
Summary of risk of bias.

Two studies of this review were marked as “low risk,” while one study by Kumar et al. [21] was of “high risk”, the summary of which is described in Figure 2.

## 4. Discussion

In recent years, HA has been identified as an adjuvant in various invasive and non-invasive periodontal procedures. The current review attempted to assess possible effects of administration of HA on periodontal surgery for gingival recession reduction. The impact of HA as an adjuvant in periodontal treatment was assessed using clinical criteria, with primary outcomes such as MRC and CRC, and secondary outcomes as gingival recession reduction, CAL, and keratinized tissue. Data suggested using the hyaluronic acid gel as an adjunct for better outcomes after surgical procedures.

In gingival recession reduction surgery, this systematic review revealed that application of hyaluronic acid showed a significant improvement in CRC and MRC [17,22]. The randomized clinical trial conducted by Kumar et al. showed no significant difference in complete root coverage in the experimental group (68.3%). The application of HA (gengigel 0.2%) in the trial group was made in adjunct with CAF, compared to the control group (61.6%), where CAF was used alone after 24 weeks post-operatively [21]. However, HA as an adjunct has been advocated in clinically compromised cases where better results are desired. In the trial done by Pilloni et al., a significant increase in complete and mean root coverage was observed in test groups (80% and 93.8%), respectively, when HA (2 mg/mL high-concentration gel) was applied as an adjunct in coronally advance flap surgery, compared to 73.1% of MRC and 33.3% of CRC in the control group where CAF was placed alone, 18 months post-operatively [17]. They concluded that using HA as an adjunctive agent combined with CAF is effective for treating Miller’s class I and II defects. Nandanwar et al. also observed that there was a statistically significant difference in MRC in the test group (92.93%), where HA (Hyaloss matrix) was used in adjunct with bio-absorbable membrane-like polylactic/polyglycolic acid (PLA/PGA), in comparison to the control group (84%) where sub-epithelial connective tissue graft (SCTG) was placed alone [22]. In this trial, maximum root coverage was observed, as HA was used in combination with bio-absorbable material in the test groups. Similarly, a systematic review conducted by Cheng et al. [25] suggested a combination of one or more biomaterials with HA or graft to achieve better root coverage. However, heterogeneity is observed between the studies, which may be attributed to the difference in the amount of HA applied in combination with different surgical procedures. Hence, different methodologies along with standardized protocols are required to provide evidence-based results to evaluate the efficiency of HA as an adjunctive agent in the treatment of Miller’s class I and II recession defects.

All three controlled clinical studies identified in the present review suggested additional benefits in terms of CAL and keratinized tissue gain, thus favoring application of HA along with periodontal surgery. In particular, CAL gain ranging from 1.0 to 3.03 mm was observed where HA was used in adjunct to CAF and GTR [17,22]. Similarly, in a clinical case series by Bogarede et al., in open flap debridement with adjunct application of HA for Miller’s class I or II defects, an average gain of CAL (3.8 mm) was observed 12 months post-operatively [26]. However, in the study by Kumar et al., which included 10 patients with Miller’s class I defect, HA gel application as adjunct to CAF in the test group compared to CAF alone in the control group resulted in significant gain and stability of clinical attachment levels [21]. Heterogeneity among the studies could be attributed to different graft materials used and application of HA gel. However, the result of the present review indicates that the use of HA in conjunction with CAF, GTR may provide additional clinical benefits evidenced by a further reduction in gingival recession and clinical attachment levels gain in intra-bony defects compared with CAF, GTR, and SCTG alone.

The follow-up period after initial treatment in three studies on periodontal surgery varied from 6 to 18 months, which explained the heterogeneity between them. In the Pilloni study, significant improvement in complete and mean root coverage and gingival recession reduction was observed at 18 months [17]. It was emphasized that a shorter period of follow-up time would not provide significant improvement in the clinical outcomes. Nandanwar et al. also observed a significant increase in gingival recession reduction and CRC after six months of therapy compared to baseline [22]. While in the results of the Kumar study, no significant effect of HA on outcomes after three and six months of surgery was observed [21]. Post-operative recovery time is required for tissue regeneration; in other terms, six months is not adequate time to heal. It is mentioned that better results are expected after a longer period of time [21]. In the Eliezier review, it was also mentioned that for treating intra-bony defects, HA application as an adjunctive chemotherapeutic agent provides statistically significant improvement in gingival recession reduction and CAL after 6–24 months [14]. In addition, the risk of bias assessment indicated a lower risk for Pilloni and Nandanwar studies in comparison to the Kumar study [17,21,22]. With the limitation of studies in this systematic review, the result favored Pilloni and Nandanwar studies. It could be inferred that HA application has additional benefits on mean and complete root coverage along with the reduction in gingival recession.

Other systematic reviews considered from the literature concluded that the efficacy of HA as a local chemotherapeutic agent with both non-surgical and surgical periodontal treatment was beneficial [14,27]. The Eliezer meta-analysis revealed that local application of HA, after 6–24 months of surgical procedure, there was a significant gain in CRC and CAL [14]. Our study was performed on three RCTs, and gingival reduction measured was 2.7, 1.1, and 2.5 mm, however, similar secondary objectives were also assessed in the Eliezer review, where the difference in the gingival recession reduction was 0.89 mm. This difference could be attributed to the data extraction process and studies included. Moreover, the present systematic review is done on the periodontal surgical procedure as compared to the non-surgical and surgical periodontal treatment by Eliezer et al. Data extraction was done in the last month of both the studies considered by Eliezer et al. In contrast, in the present review data was extracted at varied periods. However, due to the limitation of the available data, it is difficult to draw a conclusion on the studies’ analyses’ dependability.

Another systematic review performed by Bertl et al. focused on the benefits of HA in the non-surgical and surgical periodontal procedures [27]. This aforementioned review has included two similar RCTs as the present systematic review and concluded that HA is an adjunctive, providing desirable outcomes in terms of clinical outcomes in surgical procedures [17,21]. However, due to the heterogeneity in our review, the possibility of performing meta-analysis was negligible.

Gingival recession reduction, CRC and CAL gains, were significantly enhanced by local application of HA, as depicted in our review and the systematic reviews described above, regardless of being in non-surgical or surgical treatment. HA seemed to be a significant component in mineralized and non-mineralized periodontal tissues in molecular biology (alveolar bone, cementum, periodontal ligament). It has been found to sustain the cellular proliferation of mesenchymal stromal and pre-osteoblastic cells via regulating the BMP/Smad pathway [18]. Furthermore, because HA can accelerate tissue healing, it is highly suggested as an adjunctive in periodontal surgeries, papilla regeneration, osseointegration in dental implantation, and peri-implantitis therapies [28,29,30].

Although this systematic review had rigorous inclusion/exclusion criteria, the main limitation was including three studies and few patients to conclude desirable outcomes. Two studies [17,21] actually compared the same method with or without the addition of HA, while in one [22] study, the control group consisted of patients with a different method of coping with the recession (SCTG vs. PLA/GTR + HA). Moreover, the percentage of HA application and HA products used, different study designs (split-mouth/parallel/control trail), and follow-up period varied from 6 to 18 months among all included studies. Due to these limitations, it is challenging to draw an evidence-based conclusion. This is an important aspect to detect the methodological weaknesses of studies which might result in alteration in outcomes. Moreover, future studies should focus on appropriate methodological techniques, specific concentration and delivery systems for HA, and follow-up time to increase overall reporting quality and limit the possibility of bias.

## 5. Conclusions

Considering the limitations of this study, the available evidence suggests that using HA as an adjuvant to surgical periodontal procedures for labial gingival recession may provide additional therapeutic benefits. The role of HA in CAL gain as an adjunct to periodontal surgery in treating intrabony defects compared to surgery alone is unknown. Overall, HA as an adjunct is safe, and no adverse effects have been noted in the conducted studies.

## Figures and Tables

**Table 1:** Selection Criteria.

Participants	Healthy participants with no restrictions on age and sex who were in good general health with Miller Class I and II defects.
Interventions	Application of HA in conjunction with gingival recession surgical procedures.
Comparisons	The same surgical procedures without HA.
Outcomes	Complete and mean root coverage post-surgical treatment as primary outcome and gingival recession reduction, keratinized tissue, clinical attachment levels as secondary outcome.
Study Design	Randomized controlled clinical trials.

**Table 2:** Summary of Keywords in PubMed.

Domains	Keywords	Notes
Hyaluronic acid	(Acid, Hyaluronic) OR (Hyvisc) OR (Luronit) (Hyaluronan) OR (Hyaluronate Sodium) OR (Sodium Hyaluronate) OR (Hyaluronate, Sodium) OR (Amvisc) OR (Healon)	
Gingival recession	(Gingival Recession *) OR (Gingival Atrophy *) OR (Recession, Gingival) OR (Diseases, Periodontal) OR (Periodontal Disease)	Recession, recessions, atrophy, atrophies
Concentration	Administration and dosage	

* Indicates wild card in PubMed.

**Table 3:** Summary of Data Extraction.

Author/Year of Publication	Study Design	Participant’s Characteristics (Country, Setting, Subjects-Test (T)/Control (C))	Recession Defects	Age in Years (Mean and Range) Gender	Miller’s Classification	Index	Area of Surgical Intervention	Test HA Type	Control	Observation Time (Days)	Complete and mean Root Coverage	Gingival Recession Reduction	Clinical Attachment Level	Keratinized Tissue Gain	Adverse Effects	Author’s Conclusion
Pilloni A et al. 2019 [17]	parallel group	30/30 (15/15) Switzerland clinical setting	Gingival recession depth >2 mm	21- 41 years, 14:F, 16:M, 30 years (IQR)	Millers class I	Plaque score ≥1	Anterior teeth at least one gingival recession	HA gel+ CAF	CAF	18 months	CRC T = 93% C = 33% MRC T = 93.8 ± 13.0 % C = 73.1 ± 20.8 %	T = 2.7 [1.0] mm C = 1.9 [1.0] mm	T = 1.0 [0.0] mm C = 2.0 [0.0] mm	N/A	None	RecRe, CRC, MRC showed statistically significant results (*p* < 0.05)
Nandanwar J et al. 2018 [22]	Randomize control trails	24/24 (12/12) India clinical settings	Gingival recession depth > 2 mm	19- 37 years, 28.08 ± 5.45	Millers class 1 or II	Supra gingival	Multiple gingival recession defects on labial or buccal surfaces of the teeth	HA gel+ PLA/ GTR	SCTG	6 months	CRC T = 77.7 ± 41.03 % C = 65.2 ± 39.86 % MRC T = 92.9 ± 13.54 % C = 84 ± 21.74 %	T = 2.55 ± 0.45 mm C = 2.11 ± 0.58 mm	T = 3.03 ± 0.87 mm c = 2.35 ± 0.83 mm	T = 2.5 ± 0.53 mm C = 1.96 ± 0.54 mm	None	WKG, RecRe, MRC, CRC showed statistically significant results (*p* < 0.05) between the 2 groups.
Kumar et al. 2014 [21]	Split Mouth Design	10/10 (5/5) India clinical setting	Gingival recession depth > 2 mm	7:M, 3:F	Millers class I	Plaque score	At least one recession on premolar or canine region	HA gel+ CAF	CAF	6 months	CRC n/a MRC T = 68 ± 28.81 % C = 61 ± 30.22 %	T = 1.0 ± 0.66 mm C = 1.1 ± 0.99 mm	No difference	N/A	None	CAF+ HA shows better results as compared to control group, but it is statistically not significant.

HA: Hyaluronic acid, CAF: Coronally advanced flap, GTR: Guided tissue regeneration, SCTG: Subepithelial connective tissue graft, PLA: Polyglycolic acid T-test group, C: Control group, CRC: Complete root coverage, MRC: Mean root coverage, F: Female, M: Male, WKG: Width of the keratinized gingiva, RecRe: Recession reduction.

**Table 4:** Characteristics and Concentration of HA.

Author/Year of Publication	Characteristics and Concentration of HA
Pilloni A et al. 2019 [17]	Cross-linked HA (Hyaluronic acid, hyaDENT BG, Bioscience, Germany). The material represents a highly concentrated HA gel, is of non-animal origin, is based on a mixture of a cross-linked HA (16 mg/mL), and is a natural HA (2 mg/mL). It is characterized by a slow degradation pattern (several weeks).
Nandanwar J et al. 2018 [22]	Hyaloss matrix (HA—Esterified HA in the form of fibers, Hyaloss matrix^®^, Meta, Italy) was mixed thoroughly with a few drops of physiological saline solution in a sterile mixing container and hydrated. After hydration, hyaloss matrix was transformed into gel.
Kumar et al. 2014 [21]	Hyaluronan gel (gengigel 0.2% gel which is 0.2% hyaluronan gel marketed by Ricerfarma pharmaceuticals, Milan, Italy)

HA—Hyaluronic acid.

## Data Availability

Review was registered in PROSPERO with identification number CRD42021287145.
